# „Was uns Schizophrenie heisst …“ – Das sowjetische Schizophreniebild in den 1970er und 1980er Jahren und dessen Integration in die internationale Klassifikation der Krankheiten

**DOI:** 10.1007/s00048-023-00371-0

**Published:** 2023-11-20

**Authors:** Anastassiya Schacht

**Affiliations:** Institut für Geschichte, Universität Wien, Wien, Österreich

**Keywords:** Medizingeschichte, Psychiatrische Diagnostik, Skalen, Internationale Organisationen, Psychiatriemissbrauch, History of Medicine, Public Health, Psychiatric diagnostics, Scales, International organizations, Abuse of psychiatry

## Abstract

Der Artikel diskutiert die Erarbeitung einer wissenschaftlich kohärenten, international als widerspruchsfrei anerkannten Diagnostik milder Verlaufsformen für Schizophrenie im Lauf des 20. Jahrhunderts mit besonderem Augenmerk auf die sogenannte *blande* Schizophrenie, die in der UdSSR der 1970er und 1980er Jahre als Mittel zur politisch motivierten Einweisung von Menschenrechtsaktivist*innen herangezogen wurde. Das Argument verfolgt die *sluggish schizophrenia*, eine Schöpfung des sowjetischen Psychiaters Andrey Snezhnevsky, von ihrer Entstehung im hochproduktiven internationalen Umfeld der Expert*innen-Netzwerke des frühen 20. Jahrhunderts über die inhaltliche Entkopplung und den begrifflichen Wandel in der epistemisch isolierten sowjetischen Psychiatrie. Dieser Wandel wird auch anhand der Fallstudie mit der Internationalen Klassifikation (ICD) der Krankheiten (durch die Weltgesundheitsorganisation erlassen) veranschaulicht. Anschließend wird anhand verschiedensprachiger Ausgaben der neunten ICD-Revision der Versuch seitens sowjetischer Expert*innen rekonstruiert, international akkordierte Termini an die landesinternen politischen Gegebenheiten anzupassen und die sonst umstrittene *blande* Schizophrenie für den heimischen Gebrauch zu legitimieren.

## Abstract (en)

The article reconstructs attempts to create scientifically coherent, internationally agreed-upon diagnostics for mild forms of schizophrenia throughout the 20th century. A particular focus here lies on what became known as bland—or sluggish—schizophrenia, a particular term coined in the USSR, which became known for its frequent use in internationally contested diagnoses of human rights activists. The argument follows the diagnosis of sluggish schizophrenia from its inception in a highly productive and equally international psychiatric community of the early 20th century pioneered by prominent Soviet scholar Andrey Snezhnevsky and through its epistemic detachment and content-related transformation in the highly isolated Soviet psychiatric community since the interwar period. This transformation is analyzed with help of the case study on the International Classification of Diseases (ICD). Released by the World Health Organization, the ICD—and in particular its ninth revision—played a crucial role in the attempt to legitimize sluggish schizophrenia. The comparative study of four presumably identical ICD-passages from three languages helps reconstructing how internationally accorded terms would become adapted to the Soviet societal and political realities. The ultimate aim of the attempted adaptation, the article claims, was to provide the elsewhere contested diagnostic term “sluggish” schizophrenia with additional legitimacy per authority of the WHO and, thus, much needed credibility for domestic, and often political, use.

## Einleitung

Am 17. September wurde ich in das Dienstzimmer des Aufsichtsstaatsanwalts geführt. Hier saß eine Gruppe von Menschen, unter ihnen Lunz und die Psychiaterin, die früher mit mir gesprochen hatte.

In der Gruppe fiel mir ein grauhaariger Mann mit intelligentem Gesicht auf, der mir erklärte, daß dies die zweite Kommission des Gesundheitsministeriums sei.

„Wer sind Sie denn?“, fragte ich ihn.

„Ich bin Sneschnewski […].“^1^

„Weshalb wird eine zweite Expertise erstellt?“

„Auf Antrag des Untersuchungsrichters.“

„Das ist merkwürdig. Die Untersuchungsbehörden sind daran interessiert, mich als Schizophrenen hinzustellen […].“

„Aber auch Sie verlangen doch eine zweite Expertise?“

„Ja, eine stationäre, mit objektiven Untersuchungen, unter Teilnahme eines Psychiaters, zu dem ich oder meine Frau Vertrauen haben.“

„Die Elektroenzephalogramme beweisen nichts, die Tests ebenso wenig wie Biochemie“, sagte Sneschnewski.

Ich berief mich auf seine Arbeiten.

„Leonid Iwanowitsch, Sie beschäftigen sich ja selbst mit Methoden der Strukturanalyse. In der Psychiatrie setzt sich die Struktur aus Symptomkomplexen zusammen […]. Weshalb haben Sie kein Vertrauen zu uns? Sie kennen uns doch gar nicht!“

„Ich kenne Sie aus dem Samisdat, Nadscharow, Morosow, Sie und Professor Lunz sind ziemlich bekannt.“ (Pljuschtsch 1981: 303–304).

Leonid Pljuschtsch (1938–2015), ukrainischer Mathematiker, Dissident und somit Vertreter der staatlich verfolgten spätsowjetischen Menschenrechtsbewegung, beschreibt in der oben zitierten Szene seine wiederholte psychiatrische Begutachtung. Trotz seiner Verhaftung an seinem Wohnort Kiew wurde Pljuschtsch für die Untersuchungshaft nach Moskau gebracht. Das begutachtende Team stammte aus dem im Land wichtigsten Institut für forensische Psychiatrie, dem Serbski-Institut, kam direkt zu ihm ins Gefängnis Lefortowo und wurde von dem renommierten Professor Andrey Snezhnevsky (1904–1987) geleitet.

Seit spätestens Mitte der 1960er Jahre stand Snezhnevsky seiner Fachdisziplin als unangefochtene Leitfigur vor (Zajicek 2019: 258). Er und seine Anhänger*innen prägten die sowjetische Psychiatrie epistemisch, wissenschaftspolitisch sowie logistisch. Strategisch wichtige Stellen wurden mit nur wenigen Ausnahmen von Snezhnevskys ebenso prominenten Schüler*innen besetzt: Snezhnevsky selbst bestimmte über zwei Jahrzehnte hinweg das Redaktionsleben und die -politik der wichtigsten psychiatrischen Fachzeitschrift der UdSSR, *Korsakov*. Sein Schüler und von Pljuschtsch bereits erwähnter Anhänger Georgy Morozov leitete das Serbski-Institut und hatte laut eigener Angabe Überblick und Kontrolle über alle Diagnosen der Union (Smith & Oleszczuk 1996: 26–27). Im Gesundheitsministerium regulierte mit Eduard Babaian ein anderer Snezhnevsky-Schüler die Produktion und den Umlauf der psychopharmakologischen und suchterzeugenden Substanzen; er vertrat in dieser Kapazität auch die Sowjetunion bei Expertenkomitees der Weltgesundheitsorganisation (WHO 1961: 71–72). Auch Snezhnevsky selbst beteiligte sich an den repräsentativen Aufgaben sowie an projektbezogenen Kooperation mit der WHO:^2^ der Co-Leiter der bahnbrechenden Internationalen Pilotstudie zu Schizophrenie (IPSS) und späterer Leiter des gesamten Mental-Health-Programms der WHO Norman Sartorius verwies Jahre später explizit darauf, das Snezhnevsky eine sorgfältig auserlesene, zur Kooperation eingeladene Figur war (Sartorius 2011: 241).

Die Snezhnevsky-Schule wurde ab Mitte der 1960er Jahre vor allem auf internationaler Ebene kontinuierlich des politischen Missbrauchs von psychiatrischer Expertise, Behandlungspraktiken und Einrichtungen in der Sowjetunion beschuldigt (van Voren 2010). Eine zentrale Rolle spielte dabei die Definition einer bestimmten Subform der Schizophrenie, die zwar nicht ausschließlich, aber auffallend oft zur Diagnose – und Ausschaltung – von Regimegegner*innen herangezogen wurde (Shifrin 2017). Diese Subform – *sluggish* (auf Deutsch auch als *bland* bekannt) *schizophrenia* – wurde im von Snezhnevsky konstruierten Modell der Erkrankung ausformuliert und war in der Dynamik des international entflammten psychiatrischen Konflikts untrennbar mit seinem Werk, ihm selbst und seiner Schule als Ganzes verbunden.^3^ Die oben erwähnten Namen Morozov, Babaian, aber auch Andrey Snezhnevsky persönlich und weitere Anhänger*innen sind zumindest auf zwei Listen der in Missbrauch involvierten Psychiater*innen vertreten. Diese Listen wurden durch Netzwerke sowjetischer Menschenrechtsbewegungen zusammengestellt und außerhalb der UdSSR publik gemacht.^4^ Eine rege Zirkulation dieser Information über den Psychiatriemissbrauch verhalf dazu, den Konflikt zu einem heiß debattierten Thema im öffentlichen Diskurs und der Politik westlich des Eisernen Vorhangs zu machen sowie ihn aufs Programm der größten internationalen Organisationen im Medizinbereich zu bringen: der WHO und der Weltpsychiatrieassoziation (WPA).^5^

Der Konflikt um den sowjetischen Missbrauch traf in vielerlei Hinsicht den Nerv der komplexen Transformation, welche die Psychiatrie als Fach an sich, aber auch im gesellschaftlichen Kontext durchlief. Die Tendenz zur Popularisierung des Expert*innenwissens (Cohen-Cole 2016), die parallel zur Demokratisierung und disziplinären Öffnung der auf die Psyche bezogenen Fächer verlief (Garrabé 2003), traf auf die immer weiter zunehmende Involviertheit des breiten Publikums in Fragen der Menschenrechte beziehungsweise den Versuch, durch Aktivismus und öffentliche Kontrolle auf die etablierten politischen Strukturen und Organisationen Einfluss zu nehmen (Hurst 2017). Der Kalte Krieg fügte diesem komplexen Aushandlungsprozess unter Beteiligung der Psychiatrie eine weitere politische Dimension hinzu, indem beide Weltmächte versuchten, sich als Verteidiger der Menschenrechte (Ridder 2021) sowie Anfechter der fortschrittlichsten und somit auch humansten Wissenschaft zu profilieren (für die USA siehe z. B. Fousek 2000 bzw. Cohen-Cole 2016, für die sowjetische Positionierung z. B. Savranskaya 2008).

Im solchermaßen umkämpften internationalen Kontext legte der sowjetische Psychiatriemissbrauch für die Psychiatrie und ihre weltweiten Fachorganisationen Fragen der Expert*innenverantwortung und Autonomie schmerzhaft offen, trug zur Schärfung ethischer Richtlinien für die Psychiatrie weltweit bei und brachte sowjetische Akteur*innen in Erklärungsnot. Umkämpft während dieser Zeit war und bleibt bis heute die Frage nach Absicht und Wille missbräuchlicher Diagnosen – oder eben nach kontinuierlicher Fahrlässigkeit und Fehlleistung mehrerer Psychiater*innen, die zumindest über drei Jahrzehnte hinweg quer durch die Sowjetunion verstreut und ohne Absprache miteinander Dissident*innen als geisteskrank – eben (*bland*) schizophren – erklärten. Gerade das Modell Snezhnevskys, des leitenden Psychiaters im Land, und insbesondere die von ihm ausgearbeitete Subform *bland*/*sluggish* wurden im Lauf dieses epistemischen Konflikts zum Inbegriff des politischen Psychiatriemissbrauchs.

Der vorliegende Artikel widmet sich einer markanten, bislang kaum erforschten Episode im Lebenszyklus der *blanden* Schizophrenie nach Snezhnevsky. Wie ich im Folgenden zeige, wurde diese Schizophrenieform durch eine subtile, aber substanzielle Revision an einem international anerkannten Dokument für den landesinternen Gebrauch zusätzlich bekräftigt und legitimiert. Die Veränderung war dabei ein Bestandteil mehrerer Vorgänge, welche die international hinterfragte Glaubwürdigkeit sowjetischer Diagnostikentscheidungen im Rahmen des Schizophreniemodells Snezhnevskys im Landesinneren gegen fachinterne Meinungsdissenz zusätzlich absichern sollten.

Bei dem genannten Dokument handelt es sich um die zum damaligen Zeitpunkt neu erlassene neunte Revision der Internationalen Klassifikation der Krankheiten (ICD-9) und im Speziellen das psychiatriebezogene Kapitel V. Obwohl Russisch eigentlich eine der Arbeitssprachen der WHO war und somit automatisch auch eine russischsprachige Fassung vorlag, befand man es auf sowjetischer Seite für notwendig, das Kapitel V für die eigene landesinterne, nicht international angebundene Fachcommunity auf eine Weise anzupassen, dass die meisten Veränderungen in die Kategorie Schizophrenie (Code 295) fielen.

Mittels unauffälliger, aber substanzieller Umstellungen integrierten diese Anpassungen das komplexe, Snezhnevsky-nahe Model samt *blander* Schizophrenie in das von der WHO erlassene System. Dadurch entstand für das Zielpublikum der sowjetisch revidierten Fassung – Ärzt*innen und ärztliches Personal mit Diagnoseverantwortung – der nachhaltige und kaum hinterfragbare Anschein, als wären das gesamte Modell Snezhnevskys sowie auch die *blande* Schizophrenie ein fester, allgemein akzeptierter Bestandteil der international anerkannten Klassifikation. Die außerhalb der Sowjetunion als unpräzise und potenziell zu Missbrauch verleitend geltende und daher umkämpfte Definition wurde somit für das landesinterne Fachpublikum epistemisch legitimiert – und normalisiert. Gerade diese epistemische Normalisierung ist in Anbetracht des Zeitpunkts dieser merkwürdigen Revision – parallel zur krisenhaften Eskalation des internationalen Konflikts um den politischen Psychiatriemissbrauch in der UdSSR – kritisch einzuordnen.

Der Artikel basiert auf der Dokumentation und internen Berichten der Weltgesundheitsorganisation sowie, in Bezug auf den Psychiatriemissbrauch in der UdSSR, auf Quellen aus der Archivsammlung des Sacharow-Zentrums für demokratische Entwicklung an der Vytautas-Magnus-Universität Kaunas. Der Korpus in Kaunas ermöglicht einen umfangreichen Einblick in den Verlauf des Konflikts, die Positionierung internationaler Organisationen und deren Leitungsorgane. Die sowjetische Handlungslogik, sofern nicht durch Dokumentation der WHO, WPA sowie weiterer Akteure (meist in Kaunas einsehbar) protokolliert, muss mit Hoffnung auf bessere Verfügbarkeit der Archive in Russland sowie auch auf Deklassifizierung aktuell nicht verfügbarer Dokumente mit Vorsicht analytisch rekonstruiert werden. Zum Teil ist das mithilfe bereits öffentlich gemachter Kopien möglich – unter anderem durch das sogenannte Bukovsky-Archiv, das KGB-Dokumente zur Verfolgung der Menschenrechtsbewegung sowie gezielten Gebrauch der Psychiatrie zu diesen Zwecken belegt. All diese Bestände wurden im Rahmen der breiteren Forschung ausgiebig konsultiert und ausgewertet, treten aber wegen anders gesetztem thematischen Fokus in diesem Text notwendigerweise etwas in den Hintergrund.

## Inter-/nationale Schizophrenie

Auf die epistemische Herkunft und Validität dieses Taxons international angesprochen, gaben sowjetische Expert*innen stets an, die *blande* Schizophrenie Snezhnevskys beruhe auf den allgemein akzeptierten Theorien von Eugen Bleuler (1857–1939) und sei somit kaum etwas anderes als die auch in der Internationalen Klassifikation der Krankheiten, dem zentralen medizindiagnostischen und statistischen Leitfaden der Weltgesundheitsorganisation – in unserem Fall in der ICD‑9 –, verankerte sogenannte *latente* Form.

Als ein auf epidemiologische Statistik aufbauendes und weltweite Gültigkeit anstrebendes Projekt der WHO diente die ICD dem Wunsch nach einem Krankheitsbildersystem, das trotz der unausweichlichen Varianz in Sprachen, Nationalschulen und Diagnostikansätzen kohärente und vergleichbare Diagnosen liefern und somit im nächsten Schritt sowohl einen besseren Überblick über nationale Gesundheitssysteme ermöglichen als auch gezielte epidemiologische und therapeutische Maßnahmen erleichtern sollte. Die Weltgesundheitsorganisation führte hier seit ihrer Gründung 1948 und insbesondere beginnend mit den 1960er Jahren auch im Bereich der Mental Health einige auf transnationale und transkulturelle Vergleichbarkeit der zu diagnostizierenden Krankheitsbilder angelegte internationale Kooperationsprojekte und Studien durch (Wu 2021) – beispielsweise die *International Pilot Study of Schizophrenia* (IPSS), die 1967 anlief (World Health Organization 1967). Nebst „westlichen“ Orten wie London, Washington, D. C. oder Aarhus und osteuropäischen Metropolen wie Moskau oder Prag waren solche Projekte auch durchaus im blockfreien Globalen Süden verankert (wie etwa die IPSS mit Kooperationszentren in Agra und Ibadan; siehe World Health Organization 1973: V–IX). Dieses transkulturell und vielleicht sogar paritär angestrebte Unterfangen darf natürlich nicht isoliert von den um diese Zeit herrschenden politischen und sozialen Spannungen sowie auch unreflektiert reproduzierten kulturellen Vorurteilen betrachtet werden (Williams 2003).

Auch das Problem der fachlichen Methodologie angesichts der großen Polyphonie der epistemischen Zugänge sowie der stark variierenden Kapazitäten einzelner nationaler Gesundheitssysteme war zum Erscheinungszeitpunkt der neunten Revision der ICD ein über Jahrzehnte hinweg heiß debattiertes Thema – mit dem WHO-Bestreben, durch Studien^6^ und Tools wie etwa die PSE (Present State Examination) oder durch aufstrebende Digitalisierung, etwa mittels CATEGO, einen internationalen Konsens über die Diagnostik zu schaffen (Wu 2021: 63–94, 121–140). Diese beispiellos breite und transkulturell angelegte Basis für die neunte Revision der ICD verhalf zwar nicht zu einem universal gültigen Bild der psychischen Erkrankungen und darunter der Schizophrenie, trug aber als „Nebenerscheinung“ der weltweit angelegten Kooperationen und sich stärkenden Stellung der WHO zu einer erhöhten Vertrauenswürdigkeit und somit breiteren Akzeptanz der neuen Klassifikation bei. Bei IPSS im Spezifischen, aber auch generell waren sowjetische Gesundheitsexpert*innen an Kooperationsprojekten der WHO – und ihrem Mental-Health-Programm – aktiv beteiligt. In den 1960ern nahm auch der eingangs bereits erwähnte Andrey Snezhnevsky an Expertenkomitees für Psychiatrie teil; ein Jahrzehnt später war ein seiner Schule zuzuordnender Experte für Substanzabhängigkeiten, Eduard Babaian, aktiv involviert – mehr dazu im Folgenden. Ein späterer Generalsekretär der Weltpsychiatrieassoziation, Peter Morozov, schloss sich als damals noch junger Experte dem sowjetischen Team des WHO Mental Health Program an; seine Memoiren erwähnen mitunter auch die allgemeine Spannung und Zurückhaltung, die er wegen des größtenteils abseits der WHO tobenden Konflikts um den sowjetischen politischen Missbrauch der Psychiatrie in Genf empfand (Morozov 2016: 107–108).

Die Argumentation der sowjetischen Psychiater*innen aus der Snezhnevsky-Schule, ihr Konzept der *blanden* (bzw. *sluggish*) Schizophrenie sei aus dem internationalen Wissensbestand zur Schizophrenie entstanden, war in gewisser Hinsicht nicht falsch. Eine recht reichhaltige historische Forschung untersucht und problematisiert die Entwicklung der Schizophrenie und ihre politischen, sozioökonomischen und den Denkstil bestimmenden Diagnose-Infrastrukturen (*diagnosis infrastructures*), wie Henckes treffend formuliert (Henckes 2019: 551–554. Zu Denkstil und Denkkollektiv siehe Fleck 1999 [1935]). Diese historische Schizophrenieforschung reicht, grob umrissen, von der Zeit der erstmaligen Schöpfung der Begriffe *dementia praecox* und *schizophrenia* im Kontext der schweizerischen Forschung und zentraleuropäischer Gesellschaftsherausforderungen, Moraldynamiken und therapeutischer Ansätze (Bernet 2013) über die Erstrezeption und rasante Ausbreitung der Schizophrenie in der US-amerikanischen Psychiatrie (Noll 2011). Die Entwicklung geht weiter über den regen Wissensaustausch in der politisch immer radikaleren – und epistemisch immer biologistischeren – internationalen Wissenszirkulation der Zwischenkriegszeit.^7^ Im Rahmen der Nachkriegspsychiatrie reicht sie schließlich bis hin zu mannigfaltigen Transformationen, Öffnungen und damit verbundenen epistemischen Krisen des Fachs – und notwendigerweise der Schizophrenieforschung, die dank ihrer innerdisziplinären psychopathologischen Schwere sowie außerfachlichen Prominenz sowohl von der sich durchsetzenden Deinstitutionalisierung, vom Aufstieg der Antipsychiatrie,^8^ von der Neuorientierung und Aufarbeitung der NS-Zeit in der deutschsprachigen Expertencommunity^9^ als auch vom sowjetischen (sowie vereinzelt in anderen staatssozialistischen Ländern nachweisbaren) Psychiatriemissbrauch zu politischen Zwecken direkt betroffen war.^10^

Die rege Wissenszirkulation, die das Forschungsfeld der Schizophrenie seit den Anfängen im frühen 20. Jahrhundert prägte, sorgte für ein äußerst heterogenes Schizophreniebild bei einem Basiskonsens rund um Kraepelin und Bleuler. Auf diesem Grundkonsens mit fortbestehender nosologischer Varianz basiert auch die Schizophrenieklassifizierung in der Internationalen Klassifikation der Krankheiten.

Die ICD war als Instrument angedacht, um epidemiologische Statistiken mithilfe möglichst einheitlich verstandener diagnostischer Codes international vergleichbar zu machen. In diesem Sinne war die ICD auf ein fachliches Zielpublikum ausgerichtet: ärztliches und anderweitig spezifisch geschultes Personal, das entweder Diagnoseentscheidungen traf oder diese in Form medizinischer Dokumentation zu erfassen hatte.

Von Auflage zu Auflage veränderte sich die Platzierung sowie die Detailtiefe der Angaben zu psychiatrierelevanten Erkrankungen in der ICD, was auch die Entwicklung des medizinischen Denkens gut widerspiegelt. Die fünfte Auflage der ICD von 1938^11^ beschränkte sich auf letale Krankheiten, was für die Schizophrenie zur Folge hatte, dass nur die schwersten und auch sichtbarsten Formen beschrieben wurden: „Hebe‑, Paraphrenie; Katatonie, katatonisches Irresein, – Stupor“^12^. Alle anderen zu diesem Zeitpunkt bereits bekannten, aber milderen Formen blieben aus der Klassifikation ausgeklammert, nachdem sie zwar wesentliche Beeinträchtigungen der Lebensqualität, aber als Primärerkrankung eher unwahrscheinlich den Tod der erkrankten Person verursachten.

Abseits des Leitfadens waren diese Formen zwar in diagnostischem Gebrauch, aber oft nur lose in ihren heutigen taxonomischen Grenzen verankert, gelegentlich fließend und ineinander übergehend. Unter diesen milderen, nicht letalen Schizophrenieformen fand sich um diese Zeit auch die *latente* Schizophrenie – die für sowjetische Expert*innen am Höhepunkt des epistemischen Konflikts rund um den politischen Missbrauch des Fachs eine Art Anker für die suggerierte internationale Vergleichbarkeit der *sluggish schizophrenia* darstellte. Von Bleuler selbst beschrieben, erscheint die *latente* Form aus kritischer Sicht am ehesten als ein epistemischer Versuch, mit rückwirkender Argumentation die zu einem späteren Zeitpunkt festgestellte Erkrankung zu rekonstruieren und somit das Behandlungsprozedere zu legitimieren. Die *latente* Schizophrenie zielte auf den Start mit einer *Prodromalphase* ab – mit jener Phase also, die in allen medizinisch festhaltbaren Manifestationen der Psychose unbemerkt vorausgeht, aber anhand des erfolgten Krankheitsausbruchs retrospektiv zu rekonstruieren ist. Diese Idee der Rekonstruktion der prodromalen Phase wird im Weiteren von Bedeutung für meine Argumentation sein.

Eine weitere milde Form der Schizophrenie, die in fließendem und oft synonymem Gebrauch mit der *latenten* stand, war die *einfache*, auch *simplex *genannte Form.^13^ Otto Diem (1875–1950), der unter Bleuler promoviert hatte, formulierte in seinen Schriften ein Krankheitsbild, bei dem von einem allmählichen, jedoch nur leichten Rückgang der geistigen Fähigkeiten gesprochen wurde. Dieser Rückgang blieb laut dem Diem’schen Paradigma oft ohne weitere Progression der Symptomatik und scheint aus heutiger Sicht eher ein Konstrukt des bürgerlichen Moraldiskurses gewesen zu sein (Diem 1903: 111–112). Einen Bogen zur Sowjetunion der 1960er Jahre schlagend, ähnelten Snezhnevskys Auslegungen und exemplarische Fälle der *blanden* oder *sluggish* Schizophrenie/*schizophrenia* den Darstellungen der Diem’schen *einfachen* Form –14 was ersterem Modell zusätzliche Komplexität verlieh oder, anders gesehen, eine unzureichende Abgrenzung der Konzepte bezeugt. Dieser Umstand wurde im Lauf des Konflikts ab Mitte der 1970er Jahre sowohl durch Netzwerke von Menschenrechtsaktivist*innen beiderseits des Eisernen Vorhangs als auch von Expert*innen in den repräsentativen internationalen Organisationen WHO und WPA stets hervorgehoben und der sowjetischen Seite zur Last gelegt.

Snezhnevskys Modell der Schizophrenie war zentral, aber natürlich nicht das einzige, das die russischsprachige Psychiatriewissenschaft und Schizophrenieforschung in die Welt trug. Ein aus dem Jahr 1959 stammender Überblicksartikel Ernst Stengels für die WHO listet exemplarisch diverse koexistierende Alternativklassifikationen pro Land und kommentiert diese kurz (Stengel 1959: 601–663). Im sowjetischen Fall, wo seit 1963 die ICD zu verwenden war, führt der Überblick zwei landesübliche Schizophreniekategorisierungen auf (ebd.: 634–635): eine von Oleg Kerbikov und eine von Vladimir Gilyarovsky.^15^ Unerwähnt – möglicherweise bloß aufgrund fehlender Übersetzung – bleibt eine psychiatrische Klassifikation durch Peter Gannushkin (1875–1933), auf die sich sowjetische Psychiater*innen in ihren landesinternen Publikationen im Lauf der 1970er Jahre oftmals beriefen.

Stengels Beobachtung entging auch nicht die starke Fokussierung der sowjetischen Psychiatrie auf „*neurological and neurophysiological*“ (ebd.: 606) anstatt auf soziale Zugänge zur Ätiologie der psychischen Erkrankung als solcher. Die physiologische Fokussierung bei einem weitgehenden Ausklammern sozialer Risiken wurde auch von Nichtmediziner*innen hervorgehoben und als ein Element der Staatsideologie erklärt. Im kommunistischen Staat des neu erschaffenen *homo sovieticus* gab es der politischen Logik nach keine wirtschaftliche Ausbeutung der Bürger*innen und somit auch keine Umfeldrisiken, die das Arbeitervolk psychisch krank hätten machen können (Rostow 1954: 108–110). Die ideologisch kreierte Einengung des epistemischen Spielraums für Psyche-bezogene Disziplinen drängte somit einige Schulen und Ansätze fast gänzlich in den Untergrund (wie etwa die Psychoanalyse – siehe z. B. Etkind 2019) und formatierte andere nachhaltig um (für den Werdegang der Psychologie sowjetischer Prägung siehe Yasnitsky 2015; für die Umgestaltung und physiologisch-biologische Zuspitzung der Psychiatrie siehe Dufaud 2021). Dieselbe Neubewertung lässt sich auch anhand präferierter oder gezielt ausgeblendeter Themenbereiche verfolgen – etwa was den sowjetischen Zugang zur Homosexualität (Healey 2001), aber auch zu Selbstmorden der vermeintlich psychisch in keiner Weise betrübten Sowjetmenschen betrifft (Pinnow 2010).

Der *homo sovieticus* von der Psychiatrie beschäftigte sich auch mit der Geschichte des eigenen Fachs. Werke russischer Autoren aus dem 19. und sogar 18. Jahrhundert wurden neu gelesen und mit dem ideologischen Geschirr des sowjetischen Marxismus in Wissenschaft und Medizin zu vereinbaren gesucht (z. B. Budilova 1975).^16^ Mit vergleichbarem Erfolg und ähnlichem ideologischen Sentiment wird das Narrativ auch in der zeitgenössischen russischsprachigen Historiografie der Psychiatrie weitergetragen (etwa durch Morozov 2016).^17^

Wie Grégory Dufaud im Verlauf seiner Monografie (Dufaud 2021) nachdrücklich zeigt, wäre es viel zu vereinfachend und naiv zu behaupten, dass die sowjetische Psychiatrie nur aus einem erlaubten therapeutischen Zugang oder einer anerkannten Schule bestand. Auch der eingangs erwähnte Überblick Stengels über koexistierende Klassifikationssysteme spricht dagegen. Allerdings war es Snezhnevskys Modell, das klar dominierte – vor allem um die Zeit, als der Wissenschaftler selbst tätig war, mehreren Kommissionen und einem zentralen Redaktionsteam vom Fach vorsaß, seine Bücher für die Lehre an den größten Hochschulen der UdSSR empfohlen wurden und seine Freunde und Schüler*innen an strategisch wichtigen Posten in Ministerien und Gesellschaften platziert waren. Wenngleich Machtkonstellationen mit erkennbaren Präferenzen bei Stellenbesetzungen oder die epistemische Dominanz einer bestimmten Gruppe mit Sicherheit weltweit verbreitet sind und daher dem Staatssozialismus nicht exklusiv angelastet werden sollten, ist es gerade dieser gesellschaftliche, menschenrechtliche, politische, international gewordene Konflikt in der sowjetischen Psychiatrie, der der Dominanz von Snezhnevskys Schule eine besondere Bedeutsamkeit zuweist.

Snezhnevskys Theorie baute im Grunde also wirklich auf Vorarbeiten einiger klassischer deutsch- und russischsprachiger Autor*innen auf – das Streittaxon *sluggish* im Speziellen auf drei zentralen Quellen. Erstens integrierte dieser Begriff das Konzept der *blanden* Schizophrenie oder *schizophrenia mitis*, das Arthur Kronfeld (1886–1941)^18^ als eine besonders milde Ausprägung der Erkrankung beschrieb (Aizberg 2017). Zweitens muss hier auch die *latente* Schizophrenie Bleulers erwähnt werden, deren retrospektive Rekonstruktion wir oben kurz angerissen haben. Der dritte wichtige Impuls, der auch namensgebend war, ist auf die Werke der russisch-sowjetischen Psychiaterin Grunia Sukhareva (1891–1981) zurückzuführen, die den ursprünglichen Begriff *sluggish* – also „schleichend“ – als eine Form des Verlaufs oder des Voranschreitens dieser Erkrankung prägte. In diesem Kontext stand *sluggish* in einer Reihe mit – und war zu unterscheiden von – der chronischen, akuten oder schubhaften (episodenweisen) Schizophrenie.

Snezhnevskys Taxonomie übernahm diesen Begriff und transplantierte ihn auf eine qualitativ andere Achse: Nun war *sluggish* bei den „inhaltlich“ verschiedenen Formen verortet – wie etwa Katatonie, Hebephrenie, schizoaffektive Psychosen und so fort. Sie konnte eine Form des Krankheitsbeginns darstellen (hier der Idee Bleulers nahekommend) oder auch in der milden Ausprägung verbleiben, ähnlich zu Kronfelds *blander *Schizophrenie.

Es ist unwahrscheinlich, dass Snezhnevsky sein Schizophreniemodell und *sluggish* im Spezifischen in politischer Absicht erstellte, um gesunde Menschen für psychisch krank zu erklären, zwangsweise einweisen zu lassen und der Behandlung mit Psychopharmaka beziehungsweise gar Gewalt im Spital auszusetzen.^19^ In einem Staat mit funktionierenden *checks and balances*, mit einer politisch unabhängigen Justiz, aber vor allem in einem Staat, in dem Wissenschaft und Medizin nicht inhärent in staatliche Institutionen eingegliedert sind und die dazugehörigen Fachkräfte vom Staat selbst ausgebildet, bestellt und entlassen werden, wäre das Modell Snezhnevsky samt *sluggish schizophrenia* einfach ein weiteres nosologisches Modell – wie andere um die Zeit im deutschsprachigen Raum geläufige Ansätze von Werner Janzarik (Janzarik 1968), Peter Berner (Berner & Kisker 1972 und Berner & Kryspin-Exner 1982) oder dem DDR-Psychiater Karl Leonhard (Leonhard 1966).^20^ Allerdings trafen all diese Bedingungen auf die Sowjetunion nicht zu. Mit Dissident*innenfällen konfrontiert, standen sowjetische Psychiater*innen regelmäßig vor der schwierigen Entscheidung, entweder gegenüber dem suggestiven Kooperationsvorschlag seitens lokaler Polizei- und KGB-Stellen hin zu angebotenen Fehldiagnosen einzulenken oder vielerlei und weitreichende Unannehmlichkeiten fürchten zu müssen – bis hin zur Entlassung und strafrechtlichen Verfolgung (Koriagin 1987: 45–49).

Noch wichtiger aber war die epistemische Normalisierung, mit der auch ein geringes Abweichen von der restriktiven staatssozialistischen Norm für psychiatrierelevant und potenziell schizophren gehalten wurde. Gerade hier war die *sluggish schizophrenia* Snezhnevskys von zentraler Bedeutung. Ungebräuchlich außerhalb der UdSSR, wurde dieses Konzept Ende der 1970er durch eine gezielte Integration in die damals aktuell zu verlegende sowjetische Auflage der ICD‑9 zusätzlich legitimiert, nämlich als sei es von der internationalen Öffentlichkeit anerkannt.

Die Analyse dieser sowjetischen Übersetzung der 9. Auflage der ICD deutet auf eine deutliche und gezielte Änderung hin, bei der einige Abschnitte der internationalen Klassifikationsskala – und nicht zuletzt das Taxon *latente *Schizophrenie – an die landesüblichen epistemischen, aber offenbar auch an die politischen Anforderungen in der UdSSR angepasst wurden. Diese Änderung war subtil und ohne Vergleichsdokumente kaum sichtbar und besiegelte die Plausibilität des sowjetischen Schizophreniemodells für den landesinternen Gebrauch.

## Sowjetische Korrekturarbeit an der ICD-9

Die neunte ICD-Revision wurde im Mai 1976 in der 29. Tagung der World Health Assembly präsentiert und zur Publikation empfohlen; zum 1. Januar 1979 trat sie in Kraft (World Health Organization 1976).^21^ Zum Zeitpunkt der Erarbeitung und Implementierung waren sowjetische Medizinexpert*innen fester und auch aktiver Bestandteil der Weltgesundheitsorganisation. Unter anderem fungierte Russisch als eine der fünf Arbeitssprachen der WHO; somit lag eine von der Organisation akkordierte Leitlinie wie auch jede andere Art der offiziellen Korrespondenz und Dokumentation in WHO-akkordierter russischsprachiger Fassung bereits von Beginn an automatisch vor. Obwohl Übersetzungen und somit auch zu einem gewissen Grad anfallende Adaptationen der ICD in verschiedene Sprachen im Grunde geläufig und für die fünf offiziellen Sprachen der WHO direkt notwendig waren, wäre es doch gerade in dem Fall, in dem eine offizielle Fassung vorhanden war, naheliegend, eine größtenteils unveränderte Übernahme zu erwarten. Wo dies für andere Abschnitte der ICD‑9 stimmen mag, war es allerdings für das psychiatriebezogene Kapitel V eindeutig nicht der Fall.

Als Basis der Vergleichsstudie, die wir nun im Detail betrachten werden, dienen drei Dokumente – oder genauer gesagt drei Versionen desselben Dokuments. Zwei davon sind das jeweils in englischer und russischer Sprache verfasste *Manual of the International Statistical Classification of Diseases, Injuries, and Causes of Death*, akkordiert und erlassen durch die WHO im Jahr 1977. Das dritte Dokument ist die sowjetische Adaptation des Kapitels V (Psychische Störungen), das in der UdSSR vom Gesundheitsministerium 1983 gesondert publiziert wurde und auch heutzutage auf dem Webportal der Russischen Psychiatriegesellschaft zur Verfügung gestellt wird (Martynikhin 2014).^22^

Beginnen wir mit den zwei WHO-erlassenen Manuals. Nachdem beide Arbeitsdokumente der WHO darstellen, sollten sie auch bis auf den rein sprachlichen Faktor ident sein. Wir stoßen allerdings bereits hier auf eine unerwartete Diskrepanz. Anders als in der englischen Fassung beginnt die russischsprachige mit einem „Vorwort zur russischen Ausgabe“. Dieses Vorwort ist auf einer nicht nummerierten, dem Dokument vorangestellten Seite abgedruckt und suggeriert seiner Benennung nach, dass uns eine druckbereite Fassung der ICD für die Sowjetunion vorliegt, die auf diese Weise eingeleitet und erklärt wird. In diesem Vorwort heißt es:

Die meisten terminologischen Präzisierungen betreffen die Klasse „Psychische Störungen“, wo parallel zur Einführung einer erheblichen Zahl in russischer Sprache geläufiger Begriffe die Rubriken 312 (Verhaltensstörungen, nicht spezifiziert in anderen Rubriken), 313 (Emotionsstörungen, spezifisch für Kindheit und Jugend), 316 (psychische Faktoren, verbunden mit Krankheiten, die in anderen Rubriken klassifiziert werden) ausgelassen werden, die in der UdSSR dem Bereich Psychologie angehören.^23^

Man sollte an dieser Stelle hinterfragen, inwieweit die disziplinäre Einbettung gewisser Codes – ob in Psychiatrie oder Psychologie – ein tatsächliches Hindernis darstellen sollte, diese Codes doch weiterhin in der ICD zu führen. Die sowjetische Psychologie war durch die staatlichen Eingriffe zwar in vielen Bereichen schwächer ausgebildet als im Westen, doch überlappte sich ihr Themenbereich durchaus etwa mit dem in den USA.^24^ Es ist fraglich, ob der angegebene Grund gewichtig genug ist, um gerade das psychiatrische Kapitel auf Ebene eines WHO-Dokuments als veränderungsbedürftig darzustellen – wo die sowjetische Psychiatrie in dieser Zeit doch international herausgefordert wurde.

Abgesehen von den Codes 312, 313 und 316 stimmt der Rest des fünften Kapitels in der russischsprachigen WHO-Fassung mit dem englischen Pendant überein. Auch Schizophrenie wird in beiden gleich umschrieben – mit dem Code 295 und einer zusätzlichen vierten Ziffer von 0 bis 9. Diese neun festgelegten Formen sind recht großzügig gehalten, umfassen sie doch unter eigenständigen Codes sowohl die einfache (*simplex*) Form (295.0) als auch die *latente* (295.5), die *residuale* (295.6), „[a]ndere Formen“ (295.8) und schließlich „nicht weiter bestimmte Formen“, die die ICD vom Grundprinzip auf die Ziffer 9 auf vierter Stelle kodierte. Möglicherweise gerade wegen dieser Polyphonie milder Formen wurde in beiden WHO-Fassungen explizit darauf verwiesen, dass das Taxon *latent* aus dem Taxon *simplex*/einfache Schizophrenie ausgeschlossen wird.

Ebenso bemerkenswert erscheint dabei, dass bei *simplex* sowie auch bei *latenter* Form in der Kurzanleitung explizit davon abgeraten wird, diese Formen in der diagnostischen Praxis allzu bereitwillig, wenn überhaupt, zu nutzen. Auch beim Taxon 295.9 „ohne nähere Angabe“ warnen die zwei WHO-Fassungen explizit, die Rubrik solle nur „als letzte Möglichkeit“ genutzt werden.^25^

*Sluggish* – also die mild progrediente Schizophrenie Snezhnevskys – kommt weder in der englisch- noch in der russischsprachigen Version des WHO-Manuals vor. Während das für die englischsprachige Fassung zu erwarten war, überrascht das Ausbleiben dieser Form in der russischen Übersetzung doch.

Eine mögliche Erklärung wäre zum Beispiel ein Übersetzungsfehler, sollten wir etwa kontraintuitiv annehmen, dass die Weltgesundheitsorganisation ihr Dokument in einer der offiziellen Arbeitssprachen ohne Mitarbeit von Spezialist*innen mit dem notwendigen fachkundlichen Wissen der landesüblichen Begrifflichkeiten für Psychiatrie übersetzen lassen würde. Nachdem uns aber die sowjetische Ausgabe aus dem Jahr 1983 ebenso vorliegt, kann diese Hypothese leicht überprüft und negiert werden (Gesundheitsministerium der UdSSR 1983). Sollte sie stimmen, müsste die Version von 1983 schließlich auch bei vielen anderen Textstellen – bei Taxennamen sowie deren Erläuterung – essenzielle Unterschiede aufweisen.

Vergleichen wir allerdings etwa den Einführungstext für Code 295 Schizophrenie, müssen wir festhalten, dass er von der WHO zur sowjetischen Adaptation kaum geändert wurde. Auf Passagen mit wortwörtlicher Übernahme folgen einige Stellen, wo Sätze zwar in der Form unverändert blieben, jedoch in der Reihenfolge getauscht wurden. Gelegentlich teilte man einen Satz und schob den zweiten Teil einen Satz weiter nach hinten oder änderte die Schaltworte und Wortverbindungen minimal ab. Dieselbe Kongruenz – oder eher Textübernahme – ist auch an weiteren Stellen quer durch das Kapitel verfolgbar. Somit ist es unwahrscheinlich, dass der Begriff *sluggish* einer unbedarften Übersetzung durch nicht-Psychiater*innen auf WHO-Ebene zum Opfer fiel.

Eine andere mögliche Erklärung wäre, so wie von sowjetischen Psychiater*innen der Schule Snezhnevskys oftmals behauptet, dass die Begriffe *latent* und *sluggish* in dem Land vollsynonymisch verwendet wurden.^26^ Diese Annahme würde die Abwesenheit von *sluggish* etwas besser erklären. Zur Überprüfung dieser These kann die sowjetische Ausgabe von 1983 hilfreiche Auskunft geben.

Die mild progrediente Schizophrenie begegnet uns in dem Dokument von 1983 zum ersten Mal an der Stelle, wo sowohl die englische als auch die russische WHO-Fassung die *latente* platzieren: in der Exklusionsklausel beim Taxon *simplex*. Wo der „internationale“ russische Text der WHO für die bessere Abgrenzung und den Ausschluss der *latenten* Schizophrenie aus dem *simplex*-Code plädiert, behauptet die UdSSR-intern ausgerichtete Auflage von 1983, ausgeschlossen werde die „mild progrediente simple [Hervorhebung im Original]“ Form mit dem Code 295.52. Diese Formulierung erscheint sonderbar, suggeriert sie doch, dass abseits des synonymischen Gebrauchs auch weitere, quasi „unsimple“ Formen vorhanden seien. Außerdem fällt die fünfstellige Kodierung auf, wo im Originaltext der WHO nur mit vier Stellen kodiert wurde.

Auch die Benennung des Taxons 295.5 liest sich in dieser sowjetischen Fassung von 1983 anders als in der russischsprachigen WHO-Version von 1977: „*Latente Schizophrenie (sluggish, mild progredient)*“^27^. Eine nähere Lektüre legt weitere Textänderungen taxonomischer sowie nosologischer Natur offen.

Die Tab. 1 bietet eine Gegenüberstellung des Codes 295.5 aus den drei analysierten ICD-9-Fassungen. Waagerechte Zeilen führen dabei die sinntragende Funktion, die entsprechende Textabschnitte quer durch die drei Versionen innehaben oder auslassen.

**Table 1:** 

	ICD‑9 EN (WHO, 1977a)	ICD‑9 RU (WHO, 1977b)	ICD‑9 RU (Gesundheitsministerium der UdSSR 1983)
*Titel des Codes:*	Latent	Latent	Latent, sluggish, mild-progredient
*Präambel zur allgemeinen Beschreibung des Taxons*	It has not been possible to produce a generally acceptable description for this condition	Mit dem englischen WHO-Text ident.	Fehlt
*Hinweis auf empfohlene Nichtanwendung*	It is not recommended for general use, but a description is provided for those who believe it to be useful:	Mit dem englischen WHO-Text ident.	Fehlt
*Allgemeine Beschreibung der taxonrelevanten Symptomatik*	… a condition of eccentric or inconsequent behaviour and anomalies of affect …	Mit dem englischen WHO-Text ident.	Mit dem englischen WHO-Text ident.
*Wiederholter Hinweis auf nur schwierige Abgrenzung und Diagnostik*	… which give the impression of schizophrenia though no definite and characteristic schizophrenic anomalies, present or past, have been manifest	Mit dem englischen WHO-Text ident.	Fehlt
*Rechtfertigung der Platzierung und wiederholte Warnung*	The inclusion terms indicate that this is the best place to classify some other poorly defined varieties of schizophrenia	Mit dem englischen WHO-Text ident.	Fehlt
*Beschreibung der Symptomatik* (NB: nur sowjetische Fassung von 1983)	Fehlt	Fehlt	„Nicht selten für das Krankheitsbild sind pseudoneurotische, Psychopathie-ähnliche, paranoiale Störungen. Die angeführten Störungen können sowohl permanent, dauerhaft, als auch episodisch sein“
*Code-relevante Symptomatik und gleich zu kodierende Formen:*	Latent schizophrenic reaction. Schizophrenia: – borderline – prepsychotic – prodromal Schizophrenia: – pseudoneurotic – pseudo psychopathic	Mit dem englischen WHO-Text ident.	Mit dem englischen WHO-Text ident.
*Sluggish:* (NB: nur sowjetische Fassung von 1983)	Fehlt	Fehlt	„*Sluggish schizophrenia:* pseudoneurotische, Psychopathie-ähnliche, simple, paranoide (paranoiale)“
*Auszuschließende Formen:*	Excludes: schizoid personality (301.2)	Mit dem englischen WHO-Text ident.	„Ausgeschlossen: schizoide Psychopathie (301.2)“
*Sluggish-Ausführung mit fünfstelliger Kodierung:* (NB: nur sowjetische Fassung von 1983)	Fehlt	Fehlt	„295.51. *Sluggish* mit pseudoneurotischer, Psychopathie-ähnlicher Symptomatik 295.52. *Sluggish* simple Schizophrenie 295.53. *Sluggish* paranoide (paranoiale) 295.54. Latente Schizophrenie 295.59. *Sluggish schizophrenia* o. n. A.“

Dabei wird sichtbar, dass, während die zwei WHO-Fassungen erwartungsgemäß inhaltlich übereinstimmen, die sowjetische Fassung von 1983 den Code 295.5 in einer sehr stark überarbeiteten Form darlegt. Sie verändert im Grunde den gesamten Codeabschnitt. Aus dieser Fassung verschwindet sowohl die Präambel über die Unmöglichkeit, eine allgemein annehmbare Beschreibung für das zu definierende Taxon zu finden, als auch die Warnung vor dem Gebrauch, die die WHO-Texte explizit anführen. Anstelle der starken Position tritt daher im Textstück von 1983 das, was in den anderen drei Versionen lediglich ein Nebensatz war: die allgemeine Symptomatik des Taxons 295.5. Ohne einleitende Einschränkung und Nicht-Empfehlung suggeriert eine solche Darlegungsart, der Code 295.5 – ob *latent* oder *sluggish* – sei eine ganz übliche, reichlich beschriebene Form, die an keiner Stelle aufgrund unpräziser Formulierung und nur schwer definierbarer Klassifikationsgrenzen problematisch – oder gar in der internationalen Expert*innen- und breiteren Öffentlichkeit wegen dem Potenzial zum politischen Missbrauch angefochten – sei.

Auch die Fortsetzung, die in allen anderen Fassungen vom „Eindruck der Schizophrenie“ beim Ausbleiben manifester Symptomatik spricht und somit erneut die begriffliche Unklarheit hervorhebt, verschwindet aus der Version von 1983. Mit ihr fällt zudem der Abschlusssatz der Einleitung weg, in dem die Schlussfolgerung über die Nicht-Empfehlung und der Hinweis formuliert werden, dass das Taxon anderswo nicht einzuordnende Formen versammelt. Die sowjetische Ausgabe von 1983 relativiert hingegen in keiner Form und setzt mit weiteren Details zum Krankheitsbild der sowjetischen Prägung fort:

Nicht selten für das klinische Bild ist das Vorhandensein der pseudoneurotischen, psychopathie-artigen, paranoialen Störungen. Die aufgelisteten Störungen können sowohl permanent und dauerhaft sein als auch episodisch [auftreten].^28^

Auf diese Weise präsentiert, weicht der Code 295.5 deutlich von dem Bild ab, das seine Pendants in den von der WHO erlassenen Versionen vermitteln. Jegliche Relativierung der Begriffsplausibilität ist ausgeschlossen. Aus einem kurzen Nebensatz wird der Aufhänger für den ganzen neuen Paragrafen, der die ohnehin fließende Grenze zwischen dem Subklinischen und dem Schizophrenie-relevanten noch breiter und für anderswo nur bedingt relevante Symptome – oder etwa für gesellschaftsuntypische Aktivitäten aus der Spannbreite des politischen Aktivismus – als diagnostikrelevant pathologisiert. Auch die weitere Ausführung an dieser Textstelle lässt keinen Platz für Zweifel an der allgemeinen Gebräuchlichkeit des Codes. Zudem wird hier die Bestehensdauer der Symptomatik explizit angesprochen. Mit der Behauptung, dass an sich milde, wie eine Neurose anmutende Symptome nicht unbedingt dauerhaft, sondern auch nur episodisch auftreten können, wird das Deutungsfeld noch breiter und inklusiver.

Die Bezeichnung *paranoial*, die in diesem Paragrafen genutzt wird, taucht in keiner der anderen Fassungen auf – auch nicht in der russischsprachigen der WHO. Dieser Begriff errang seinen Ruhm im Kontext der politischen Psychiatrie, wurde er doch zur Beurteilung einiger Dissident*innen herangezogen. Alexander Voloshanovich, ein in den Westen geflüchteter Menschenrechtsaktivist und selbst Psychiater, erklärte diesen Begriff auf Anfrage als einen nirgends außerhalb der Sowjetunion anerkannten Terminus.^29^ Ein anderes Dokument der sowjetischen Psychiatrie, der *Methodische Bref* – eine aus dem Jahr 1970 stammende Unterweisung für medizinisches und forschendes Personal, welche Symptomatik als schizophren anzusehen ist – definierte *paranoiales* Verhalten als solches, bei dem die erkrankte Person

[…] eine/die „gerechte“ Sache [Anführungszeichen gemäß Original] verfolgend, […] endlose Beschwerden schreibt, die eigene Korrespondenz pedantisch sammelt und archiviert, intensiv Tagebuch schreibt […] (Zharikov & Liebermann 1970: 55–56).

Diese Beschreibung würde dem Begriff der Querulanz nahekommen, wäre sie nicht in einem System gebräuchlich, in dem Meinungsabweichung pathologisiert und kriminalisiert wurde, während die Opposition auf gewaltfreie Protestformen setzte – wie etwa das Abfassen von Beschwerdebriefen und Ähnliches. Mit der Mitaufnahme des *Paranoialen* in die Beschreibung von Code 295.5. der sowjetischen ICD-9-Version wurde auch diese Art des friedlichen Protests pathologisiert.

Es blieb allerdings nicht beim Paranoialen. Es sind insgesamt fünf Formen, die keine der oben diskutierten internationalen Fassungen der ICD‑9 aufführt und die hier unter dem Namen der ICD als von ihr anerkannte Formen dargestellt werden. Jede dieser Formen erhielt in der sowjetischen Fassung von 1983 eine weitere, fünfte Kategorisierungsziffer, wobei tatsächlich vier davon inhaltlich besetzt wurden und der Subcode 259.59 o. n. A. – „ohne nähere Angabe“ – blieb. Somit sollte er in einem ohnehin schon lose aufgebauten diagnostischen Taxon mit bereits mehreren „Sammelbecken“ eine weitere Stelle zum Versammeln schlecht formulierbarer Diagnosevermutungen sein.

Es ist ebenfalls von Bedeutung, welche Bezeichnung diese fünf Formen erhielten, die in der sowjetischen Fassung von 1983 auftraten. Oben habe ich die Vermutung geäußert, das Ausbleiben des Terminus „mild progredient“ aus der internationalen Version der ICD beruhe darauf, dass dieser Begriff im sowjetischen Gebrauch mit dem Begriff *latent* synonymisch benutzt werde. Zu einem gewissen Grad bestätigt sich diese Überlegung anhand der Überschrift für den Code 295.5, wo beide Formen nebeneinander angeführt werden. Die fünfstelligen Codes zeigen aber eine merkwürdige Aussonderung des Begriffs *latent* in eine eigene, mit keinen weiteren präzisierenden Attributen versehene Form:

295.51. Mild progrediente Schizophrenie mit pseudoneurotischer und pseudopsychopathischer Symptomatik.

295.52. Mild progrediente simple Schizophrenie.

295.53. Mild progrediente paranoide (paranoiale) Schizophrenie.

295.54. Latente Schizophrenie.

295.59. Mild progrediente Schizophrenie o. n. A*.*^30^

Dies suggeriert, dass zumindest auf der konzeptuellen Ebene sehr wohl zwischen *latent* und *sluggish* unterschieden wurde und das Hauptinteresse und daher auch die meisten Präzisierungen gerade Letzterer galten, während die Erstere aus der Verpflichtung zu internationaler Vergleichbarkeit heraus erwähnt wurde. Das Integrieren des rein sowjetischen – zu dem Zeitpunkt auch aufgrund des politischen Missbrauchs mehrfach angefochtenen – Taxons *sluggish* erscheint hier als willentlicher Versuch, diesen Begriff für den internen Gebrauch zusätzlich abzusichern.

Das sowjetische Fachpublikum, das mit großer Wahrscheinlichkeit nur die russischsprachige Version der ICD lesen konnte, erhielt unter dem Namen des international anerkannten Kategorisierungssystems ein beachtlich umgearbeitetes Kapitel. Abseits von Code 295 bestanden weitere Veränderungen in der Formulierung und im Wegschneiden einiger Cluster – etwa in Bezug auf Substanzabhängigkeiten, was „im Einklang mit den prinzipiellen Einstellungen und Anforderungen der vaterländischen Psychiatrie“^31^ geschah.

Die Abänderung des Codeabschnitts zur Schizophrenie besaß allerdings diese zusätzliche Dimension des internationalen Konflikts rund um den politischen Missbrauch sowjetischer Psychiatrie an Dissident*innen. Die breite und institutionell verankerte Praxis von Fehldiagnosen bestand bereits vor der Revision. Auch die bestehende Diskrepanz der landesüblichen Begrifflichkeiten wie *sluggish* zu denen abseits der UdSSR geriet vereinzelt ins Blickfeld der internationalen Expert*innencommunity – etwa im Fall der International Pilot Study of Schizophrenia, von der WHO initiiert und beaufsichtigt. Die IPSS selbst ist in den letzten Jahren zunehmend zum Objekt medizin- und wissenschaftshistorischer Forschung geworden, wie etwa durch Charmaine C. Williams’ Analyse der Expert*inneninterpretation der Daten aus „developed“ and „developing“ Ländern, bei der die besseren epidemiologischen Angaben aus den Letzteren implizit so umgedeutet werden mussten, dass sie die diskursiv dominante Rolle des Westens nicht herausforderten (Williams 2003). Die Daten aus der UdSSR wurden bei der IPSS wohl zu denen aus „entwickelten“ Ländern gezählt, wobei die Studie etwas höhere Inzidenzen in der Sowjetunion und den USA auf die jeweils landesüblichen breiteren Schizophrenie-Auffassungen zurückführte.

Der Bericht aus dem Jahr 1973 erwähnt auch, dass das Moskauer Kooperationszentrum Patient*innen nach „der eigenen Terminologie des Zentrums“ beurteilte, die von jener an allen anderen Standorten der Studie abwich;^32^ darunter fiel auch die *blande* (*sluggish*) Schizophrenie, die man mangels besserer Verortung mit der *latenten* zusammenfallen ließ.^33^ Allerdings hebt das Dokument diese vorhandene Divergenz zwischen den sowjetischen und allen anderen Nomenklaturen der Schizophrenieformen hervor, und die Ersteren erscheinen dabei eindeutig nicht ICD-konform.

Während es in der ersten Hälfte der 1970er für sowjetische Expert*innen noch möglich gewesen war, die Fachdebatte so weit zu kontrollieren, dass begriffliche Unstimmigkeiten international nicht beanstandet wurden und Missbrauchsfälle weder im Land noch international zu breiteren Diskussionen unter Psychiater*innen führten, wurde dies mit der Zeit immer schwieriger. International geriet man im Verlauf des Jahrzehnts zunehmend unter Druck, wobei nun nicht nur Medien und Menschenrechtsgruppen, sondern auch Fachleute sowjetische Diagnosen öffentlich in Zweifel zogen. 1977 sprach die Generalversammlung der WPA auf ihrem 6. Weltpsychiatriekongress eine scharfe Verurteilung aus, in der die politisch motivierten Praktiken vonseiten der sowjetischen All-Union Gesellschaft der Neurolog*innen und Psychiater*innen von der Generalversammlung offiziell und öffentlich herausgefordert wurden. Was nur ein paar Jahre zuvor, wie etwa im Fall der IPSS, noch halbwegs durchgegangen war – etwa die Plausbilität fraglicher Diagnostikentscheidungen mit divergierenden Klassifikationstraditionen zu untermauern –, war gegen Ende der 1970er Jahre kaum mehr möglich.

Eine Chance der Sowjetunion, den Konflikt vor einer weiteren Eskalation zu bewahren, lag im Einwirken auf Expert*innen im eigenen Land. Wenn auch nur vereinzelt, gab es im Lauf des Jahrzehnts doch einige Psychiater*innen, die die herkömmlichen Diagnosepraktiken hinterfragten beziehungsweise in einigen namentlich bekannten Fällen auch aktiv gegen politisch motivierte Diagnosen vorgingen und mit Menschenrechtsgruppen kooperierten. Gegen solche „abtrünnigen“ Kolleg*innen wie Alexander Voloshanovich (*1941), Marina Voikhanskaya (*1934), Semion Gluzman (*1946) oder Anatoly Koriagin (*1938) wurde mit Entlassung, Zwangsausbürgerung oder Haftstrafen vorgegangen.

Eine etwas schonendere Containment-Strategie in Russland selbst wäre aber denkbar, wenn jeder Zweifel an sowjetischen Diagnoseentscheidungen oder deren Überlappung mit internationalen Richtlinien im Fach beseitigt werden könnte. Während die Glaubwürdigkeit der sowjetischen Mainstream-Klassifikation für Schizophrenie nach Snezhnevsky international herausgefordert wurde, sollte sie zwecks landesinterner Kohärenz und Compliance möglichst breit legitimiert und gestärkt werden. Gerade dafür bot die um die Zeit anfallende Revidierung der ICD eine günstige Gelegenheit. Eine ICD mit an Snezhnevsky angepasste Terminologie minimierte jeden potenziellen Meinungsdissens unter sowjetischen Fachleuten.

Seit dem Erlass des sowjetischen Kapitels V innerhalb des Landes war es kaum mehr möglich, die Plausibilität der Snezhnevsky’schen Taxonomie der Schizophrenie und von *sluggish* zu hinterfragen. Während man sich im Zweifelsfall internationaler Richtlinien – ergo der ICD – hätte bedienen können, erzeugte die Fassung von 1983 den Anschein, dass die internationale medizinische Gemeinschaft – und somit auch die WHO vertreten durch die ICD – *sluggish* als diagnostische Einheit anerkannte. Um die letzten Zweifel auszuräumen, postulierte auch das Vorwort zum gesamten Kapitel V, dass zwar erhebliche Anpassungen gemacht wurden, aber „ohne wesentliche Veränderungen nur folgende Rubriken geblieben [sind]: Schizophrenie (295.–)“.^34^

Ein sowjetischer Psychiater oder eine Psychiaterin hätte also auch im Zweifelsfall weder eine Chance noch einen Grund – sowohl wegen der Sprachbarriere als auch wegen der nur eingeschränkten Verfügbarkeit nichtsowjetischer Publikationen –, an der im Land herrschenden Taxonomie zu zweifeln. Für eine sowjetische Fachkraft war und blieb die *sluggish*, mild-progrediente Schizophrenie ein feststehender Bestandteil der Ausbildung für den Beruf sowie der alltäglichen beruflichen Sozialisierung: In Moskau gedruckte Fachliteratur und die aktuelle Forschung zu Schizophrenie in der Zeitschrift *Korsakov* verwies auf Snezhnevsky. Die eigens veranlasste Integration in die ICD verlieh dem Modell zusätzliche Glaubwürdigkeit, welche das System angesichts des Drucks von innen durch Dissident*innenbewegungen und von außen durch nichtstaatliche Organisationen, Politiker*innen und Kolleg*innen (Hurst 2017) dringend benötigte. Ein so hohes Maß an Glaubwürdigkeit konnte nur der international gültige Leitfaden der Weltgesundheitsorganisation besitzen.

## Das zuständige Team

Wer waren aber die konkreten Akteur*innen, die die massive Änderung des Kapitels V in ICD‑9 für die Sowjetunion veranlasst und durchgeführt hatten? Die Version des Kapitels von 1983 listet 37 Mitwirkende auf und führt auch ihre Affiliationen an, was uns einen guten Einblick in die Entscheidungslogik hinter diesem Dokument ermöglicht.

Die Revision wurde laut Titelblatt von zwei zuständigen Stellen des sowjetischen Gesundheitsministeriums betreut und publiziert: von der Abteilung für Medikamente und medizinische Technik und von jener für medizinische Statistik und Rechentechnik.

Der Leiter der letztgenannten Abteilung, Grigory Tserkovny, vertrat als Haupt eines vierköpfigen Teams auf der internationalen Konferenz zur neunten Revision der ICD, die Ende September 1975 in Genf tagte, die Sowjetunion – und war Co-Leiter ebendieser Konferenz.^35^ Die restlichen drei Mitglieder seiner Delegation formten ein Team für das regionale Zentrum zur Klassifikation der Krankheiten, das am Semashko-Institut in Moskau verankert und mit der Koordination der aktuellen ICD-Revision für den russischsprachigen Raum betraut war.^36^

Es ist naheliegend, dass dieses Team die russische WHO-Fassung sowohl auf internationaler Bühne als auch in der Sowjetunion betreute. Ebenso plausibel ist, dass es am Semashko-Institut die Überprüfung der Kompatibilität ihrer WHO-Version mit landesüblichen Begrifflichkeiten an Kolleg*innen in Einzeldisziplinen wie der Psychiatrie delegierte. Ob dies auf eigene Initiative geschah oder inoffiziell gesteuert wurde, lässt sich anhand der Dokumentenlage nicht rekonstruieren. Allerdings ist sehr wahrscheinlich, dass die Abänderung der ICD-Revision gerade an dieser Delegation ansetzte.

In diesem Zusammenhang betritt die zweite erwähnte Abteilung des Gesundheitsministeriums die Bühne, die in die Adaptationsarbeit involviert war – diejenige für Medikamente und medizinische Technik. Auf den ersten Blick scheint es, als hätte sie mit der Implementierung der ICD – eines statistischen Instruments der medizinischen Diagnostik – thematisch eher wenig zu tun. Diese Abteilung unterstand allerdings der Leitung einer äußerst interessanten Person, die nach außen mit der WHO und nach innen sowohl mit dem Gesundheitsministerium als auch mit weiteren prominenten Forschungs- und Behandlungszentren affiliiert war: Eduard Babaian. Diese Tatsache und die medizinische Spezialisierung auf Psychiatrie lassen vermuten, dass er eine Schlüsselrolle beim Weiterreichen und der Transformation der ICD‑9 in der Sowjetunion spielte.

Babaian, ein Psychiater mit Schwerpunkt auf Narkologie und Pharmakologie, gehörte innerhalb der Sowjetunion zur Moskauer Schule rund um Andrey Snezhnevsky und somit auch zu jenen wenigen hochrangigen Expert*innen im Fach, die sowohl prestigeträchtige und politisch entscheidende Schnittstellen besetzten als auch mit der internationalen Repräsentation betraut waren. Babaian vertrat sein Land bei der WHO: Ab dem Jahr 1962 taucht sein Name auf der Liste der Mitglieder des Expert*innenratskomitees für die Organisation der Gesundheitspflege,^37^ zehn Jahre später im Komitee für Drogenabhängigkeit auf.^38^ Aus der gemeinsamen Arbeit im Gesundheitsministerium sowie bei der WHO musste Babaian auch Tserkovny persönlich gekannt haben.^39^

Babaian erwies sich außerdem als aktiver und staatsloyaler Akteur, da er 1977 an der Seite des stellvertretenden Gesundheitsministers Dmitry Venediktov (im Amt von 1965 bis 1981) die sowjetische Delegation zum VI. Weltpsychiatriekongress in Honolulu leitete – ausgerechnet bei dem Kongress also, wo der seit einigen Jahren wachsende Konflikt rund um den Psychiatriemissbrauch in der UdSSR in Form einer offenen Konfrontation ausgetragen wurde, die sowjetische Delegation heftiger Kritik ausgesetzt war und anschließend einer kollektiven Verurteilung durch die Resolution der Großversammlung unterzogen wurde. Unmittelbar nach dem Kongress gab Babaian in Genf eine Pressekonferenz, die zur wiederholten Verteidigung der sowjetischen Psychiatrie dienen sollte (Jurt 1977), während der oben erwähnte stellvertretende Minister Venediktov, zugleich ein sowjetischer Vertreter in der WHO, kaum eine Woche später beim Treffen des europäischen Regionalkomitees eine aufflammende Diskussion zum Missbrauchskonflikt und der soeben erlassenen Verurteilungsresolution der Weltpsychiatrieassoziation blockierte.^40^ Babaian gehörte gemeinsam mit Venediktov und Snezhnevsky zu den Autoren eines offiziellen Berichts – beziehungsweise eines darin enthaltenen Planungsentwurfs für spätere Aktivitäten gegen die internationale Ausbreitung des Konflikts –, den sowjetische Psychiater*innen nach dem Weltkongress in Honolulu an das Zentralkomitee ablieferten.^41^ Mit einem weiteren Kollegen aus dem engsten Kreis um Snezhnevsky, Georgy Morozov, betreute Babaian im August desselben Jahres schließlich eine Delegation österreichischer Psychiater*innen in der UdSSR, die die ethische Tadellosigkeit und die epistemische Validität der sowjetischen Psychiatrie für die internationale Öffentlichkeit bezeugen sollte.^42^ Morozov bekleidete den Posten des Leiters einer für die Sowjetunion fachlich einzigartigen Institution – dem Serbsky Institut für forensische Psychiatrie, das im Konflikt um den politischen Missbrauch als die erste und zentrale Stelle für Fehldiagnosen bekannt war. Sowohl Babaian als auch Morozov waren also prominente Experten, international sowie landesintern fachlich renommiert und auf politische Loyalität gegenüber dem Regime hin erprobt – und beide waren in die Vorbereitung der sowjetischen Druckauflage der ICD 1983 involviert.

Unter den insgesamt 37 in der Druckfassung von 1983 namentlich aufgelisteten Mitwirkenden waren drei mit Babaians Abteilung am Gesundheitsministerium affiliiert (überprüfbar auch über Central Intelligence Agency of the United States of America 1975). Drei weitere kamen aus der Statistischen Abteilung Tserkovnys (und niemand aus dem der WHO bekannten Team). Diese sechs „ministerialen“ Namen werden erst ganz am Ende der Autor*innenliste angeführt ohne zu präzisieren, worin genau ihr inhaltlicher Beitrag zur Adaptation bestand – höchstwahrscheinlich fungierten sie in erster Linie als eine koordinierende Schnittstelle zwischen den internationalen und den landesinternen Expert*innen. An Letztere, auch vom Hauptfach her Psychiater*innen, muss man die eigentliche Anpassungsarbeit delegiert haben.

Diese restlichen Mitwirkenden lassen sich bis auf zwei Personen anhand ihrer Affiliation in zwei große Gruppen aufteilen. Die erste Gruppe umfasst Mitwirkende aus dem Psychiatrieinstitut der Akademie der Medizinischen Wissenschaften der UdSSR. Dasselbe Institut ist ab dem Jahr 1981 offiziell unter einem anderen Namen bekannt: Forschungszentrum für Psychische Gesundheit (Maximov u. a. 1984: 72). Es war eine hochspezialisierte Einrichtung, die zum damaligen Zeitpunkt seit einigen Jahren der Leitung Andrey Snezhnevskys unterstand.

Insgesamt acht Expert*innen, die an der Vorbereitung der ICD-Adaptation mitwirkten, waren mit diesem Institut affiliiert. Einige von ihnen hatten bereits Erfahrung mit Kategorisierungsleitfäden. So verfassten 1970 zwei auch hier beteiligte Forscher, Juri Liebermann und Nikolay Zharikov, einen sogenannten *Methodischen Brief* (Zharikov & Liebermann 1970). Diese Einweisung fasste zusammen, nummerierte und kodierte 76 „Syndrome“, die für verschiedene Manifestationen der Schizophrenie charakteristisch sein sollten.

Nur fünf dieser „Syndrome“ wurden dabei eindeutig an bestimmte Formen der Schizophrenie geknüpft. Die restlichen Manifestationsarten stellen eine Mischung aus mehr oder weniger kohärent erscheinenden Symptombeschreibungen dar. Für die medizinhistorische Forschung ist dieses Dokument unter anderem auch als Argumentationsbeispiel interessant, das für diagnoseverantwortliche Fachkräfte einen sicheren Diskussions- und Handelsraum für Hyperdiagnostik darbot, wenn notwendig. Einer unter Zuhilfenahme des *Methodischen Briefs* von Liebermann und Zharikov zu begutachtenden Person konnte zum Beispiel anhand eines – nach subjektivem Urteil der Gutachter*in – vermeintlich zu großen Interesses an Philosophie oder modernistischer Kunst eine „metaphysische Intoxikation“ attestiert werden (Syndrom 56 in Zharikov & Liebermann 1970: 49–50), die als symptomatisch für sowjetische Schizophreniekonzepte zählte.

Zharikov sammelte außerdem gemeinsam mit seinem ebenso im Redaktionskollegium vertretenen Kollegen aus dem Kreise Snezhnevskys, Ruben Nadzharov, seine international verwertbare Erfahrung bei der oben kurz angeführten, unter dem Schirm der WHO Mitte der 1970er Jahre durchgeführten Internationalen Pilotstudie zur Schizophrenie (IPSS). Mit dem Kollektiv aus Zharikov, Nadzharov und Liebermann hatte die die ICD revidierende Runde also auf jeden Fall drei bereits gut erprobte, in der breiten Anwendung der Begrifflichkeiten erfahrene Forscher. Zharikov und Nadzharov werden namentlich in der Liste der sowjetischen Expert*innen erwähnt und können direkt, durch institutionelle Begünstigung oder durch öffentliche Verharmlosung mit den Praktiken des politischen Missbrauchs der Psychiatrie in Zusammenhang gebracht werden.^43^

Mit 20 Personen die zahlentechnisch größte Gruppe an Mitwirkenden kam allerdings aus dem oben im Zusammenhang mit seinem Direktor Georgy Morozov erwähnten Serbsky Institut für forensische Psychiatrie. Dieses hatte in der Sowjetunion eine kontrollierende Funktion inne, indem es forensische Forschung und alle forensisch-psychiatrischen Gutachten im Land beaufsichtigte (Smith & Oleszczuk 1996: 26–27). Seit dem ersten internationalen Bekanntwerden der politisch motivierten Praktiken wurde dieses Institut stets in Verbindung mit Fehldiagnosen an Dissident*innen gebracht.^44^ Der Menschenrechtsaktivist, Buchautor und somit teilweise zeitgleicher Chronist des internationalen Konflikts um den sowjetischen Psychiatriemissbrauch Alexander Podrabinek (*1953) beschreibt in seinem Buch *Strafende Medizin* das Serbsky Institut als Inbegriff und zentralen Austragungs- sowie Entscheidungsort dieses Fachmissbrauchs (Podrabinek 1979: 72). Insgesamt fünfzehn der in der ICD-Adaptation von 1983 aufgelisteten Mitwirkenden aus dem Serbsky-Cluster waren mit dem Institut direkt affiliiert, fünf weitere mit dem Psychiatrieinstitut am Gesundheitsministerium der RSFSR, strukturell eine Serbsky-Filiale. Nicht auf den von Dissident*innen kompilierten Listen aufscheinend, aber eindeutig dem Serbsky als Institution zuzuordnen und unter der Leitung von Morozov arbeitend waren auch V. Golland, L. Miroshnichenko und A. Kiselev – alle drei als Autoren der Ausgabe von 1983 ausgewiesen.

Es lässt sich also festhalten, dass die Adaptation von 1983 in die vertrauten Hände jener Expert*innen gelegt wurde, die entweder zu den engsten Schülern Snezhnevskys gehörten, ihm dienstlich unterstanden oder aus dem Serbsky-Institut kamen, das durch den politischen Missbrauch der Psychiatrie international unter Druck stand (und eben vom Snezhnevsky-Anhänger Georgy Morozov geleitet wurde). Die Anpassung an die „landesüblichen“ Begrifflichkeiten ist also allein aufgrund der Wahl der Mitwirkenden problematisch und stand in direkter Absicht, die politisch instrumentalisierte und anschließend darauf epistemisch angefochtene Nosologie durch die von der WHO verifizierte ICD abzusichern.

## Qui prodest? – Schlussbemerkungen

Es ist aktuell nur bedingt möglich, einen tieferen Blick hinter die Kulissen der sowjetischen Entscheidungsfindung zu werfen und somit den Grad der Beteiligung einzelner Akteur*innen sowie die Absichtlichkeit hinter ihren Handlungen in erschöpfender Eindeutigkeit festzuhalten. Russische Archive sind wegen Russlands Angriffskrieg für unbestimmte Zeit nicht einsehbar. Das größere Projekt, das dem vorliegenden Text einen Rahmen gibt, konnte auf Quellenbeständen aus der Zeit vor dem Krieg und vor der Pandemie sowie auf Archivquellen aus den Ländern aufbauen, wo Prozesse der kritischen Auseinandersetzung mit der autoritären Vergangenheit nicht durch den Staat behindert werden – etwa in Litauen und der Ukraine. Eine gute Hilfe bieten KGB-bezogene Sammlungen des Wilson Center (Mitrokhin-Archiv) beziehungsweise die von Vladimir Bukovsky Anfang der 1990er Jahre aus kurzzeitig geöffneten Beständen der Staatssicherheit kopierte Unterlagen. Allerdings zeigt die Erfahrung, dass die Spur des sowjetischen Psychiatriemissbrauchs zwar bis hinauf in die Ebene der politischen Entscheidungsträger*innen rekonstruierbar ist, aber nur bedingt und rudimentär bis zu den Psychiater*innen reicht, die in dieser oder jener Rolle für Fehl- und willentliche falsche Diagnosen verantwortlich waren. In den 1970er und 1980er Jahren reflektierte die Community über die eigene Involviertheit und Motive wenn überhaupt, dann nach Möglichkeit nicht schriftlich. Die kurze Phase der kritischen Diskussion Anfang der 1990er Jahre war zwar wichtig, bezog sich aber nicht auf die ICD-Fassung von 1977 (1983), was die vorliegende Analyse potenziell der Kritik aussetzen mag als auch als wichtigen Vorstoß in Richtung einer umfangreicheren und feineren Ausarbeitung bisher übersehener Aspekte des politischen Missbrauchs der Psychiatrie in der UdSSR positioniert.

Zwar kann also nicht mit erschöpfender Klarheit festgehalten werden, ob das adaptierte Kapitel V der ICD‑9 auch von der Grundintention her eine vorsätzliche innenpolitische Spurenvertuschung darstellte oder ob dies ein mitbedachter und durchaus gewollter, aber nicht zielgerichtet beabsichtigter Nebeneffekt der auch sonst angedachten Hybridisierung der ICD‑9 in der UdSSR war. Für eine zumindest teilweise vorhandene Intentionalität sprechen meines Erachtens mehrere Faktoren. Erstens ist der Zeitpunkt der Adaptation wichtig, da angesichts des internationalen Drucks auf die sowjetische Psychiatrie eigentlich eine umgekehrte Bewegung zur Entschärfung des Konflikts nötig gewesen wäre. Eine solche Entschärfung wäre im Fall der tatsächlichen Angleichung der Diagnostik eher vorhanden als im subtilen Inkorporieren der herausgeforderten Taxa, als seien sie international akzeptiert. Zweitens sind es dieselben Akteur*innen, die an der sowjetischen Adaptation beteiligt waren und anhand der Dissident*innenquellen in Verbindung mit Missbrauchspraktiken standen. Diese Akteur*innen gehörten dem paradigmatischen und machtpolitischen Fachcluster um Andrey Snezhnevsky an – und verdankten ihre Schlüsselpositionen nicht zuletzt diesem Netzwerk.

Ebenso auffallend ist, dass das sowjetische Redaktionsteam sein Werk im Inland als Adaptation deklarierte. Das notwendige Anpassen an landesübliche Termini wurde zwar von der WHO nicht unterbunden – die neunte Revision durch die WHO trug allerdings, wie oben bereits erwähnt, drei ganz explizit ausgewiesene fachspezifische Adaptationen. Dass die sowjetische Seite eine weitere Adaptation ausgerechnet für das um die Zeit am meisten angeschlagene, skandalisierte Fach benötigte, erregt unausweichlich Zweifel. Anders als bei anderen ähnlichen Dokumenten trug dieses Kapitel V auch kein „Gütesiegel“ in Form eines Verweises auf die Betrauung zur Veröffentlichung durch die WHO.^45^ Das Redaktionsteam verschwieg auch, dass es den Schizophrenieabschnitt bearbeitet hatte und wie tiefgreifend dieser Eingriff war.

Die sowjetische Taxonomie für Schizophrenie nach Snezhnevsky barg in sich nur eine vage Abgrenzung zwischen verschiedenen Formen der Schizophrenie – der Autor verwies ja selbst auf den recht freien Umgang mit Termini wie *simplex, latent* oder *sluggish* (mild-progredient). Während diese Begriffe international nicht zuletzt wegen dieser Unschärfe graduell aus dem Gebrauch kamen, sodass in der ICD‑9 schließlich vor ihrem Gebrauch gewarnt wurde, war das in der sowjetischen Psychiatrie lange nicht der Fall.

Die überinklusive Ausweitung der schizophrenen Taxonomie, korrumpiert durch die strukturellen Eigenartigkeiten des totalitären Staatssozialismus, wurde lange vor dem WHO-Beschluss der ICD‑9 zum Mittel der Repression. International angefochten, war dieses Modell gebrauchstechnisch auf die Sowjetunion beschränkt. Durch die stark invasive Revisionsarbeit an dem 1977 durch die WHO sanktionierten Text der ICD‑9 wurde der sowjetischen Ausgabe von 1983 der Anschein einer internationalen Validität verliehen, die sie in Wirklichkeit nicht besaß.

Somit verlor durch die Revision auch die Frage nach Absicht oder Fahrlässigkeit in gewisser Hinsicht ihren Sinn. Im Endergebnis ist es nicht entscheidend, ob willentlicher Missbrauch eines diagnostischen Instruments durch erstrangige Vertreter*innen der sowjetischen Psychiatrie bei einer Begutachtungskommission im Serbsky-Institut unter Leitung Morozovs vorlag, wie im Falle Pljuschtsch am Anfang dieses Artikels erwähnt, oder eine unreflektierte Fehlleistung einer Fachkraft in einer regionalen psychiatrischen Anstalt, die womöglich bereits mit ihrem Arbeitspensum und anderen Lebenswidrigkeiten genügend ausgelastet war und dann, mit einem kniffligen Dissident*innenfall konfrontiert, nach einem Lehrbuch griff – oder eben nach der sowjetischen Fassung der ICD. Die Vertauschung machte jedes zusätzliche Nachschlagen überflüssig sowie jedes potenzielle Zweifeln an der Validität von Schizophreniediagnosen – der Schizophrenie nach sowjetischer Art.

## Footnotes

1 Bei den meisten hier erwähnten sowjetischen Akteur*innen variiert die Schreibweise ihrer Vor- und Nachnamen je nach Transliterationsvorgaben der jeweiligen Zielsprache. So findet man etwa Snezhnevsky (was in dieser Form eine englischsprachige Transliteration ist) in den Quellen auch als Sneschnewski, Sneshnewski beziehungsweise Sneshnewskij, Snieżniewski, Snezhne(v)ski, Snejnevksi usw. Ich entschied mich für die englische Transliterationsweise, um eine möglichst große Konvergenz mit den zentralen – eben englischsprachigen – Veröffentlichungen zum Thema zu gewährleisten. Im Fall Pljuschtsch behielt ich die deutsche Schreibweise bei, nachdem ich aus der gedruckten deutschsprachigen Übersetzung des Buchs zitiere.

2 Snezhnevsky war der erste sowjetische Psychiater, der mit der Aufgabe betraut wurde, die Sowjetunion im Expert Advisory Panel for Mental Health der WHO zu vertreten. Siehe dazu die interne Dokumentation der WHO: „Report on Appointment to Expert Advisory Panels and Committees by the WHO’s Executive Board of 12.01.1959“ (EB23/42).

3 Die Rolle der Snezhnevsky-Schule wird bereits um die Zeit des Konflikts von internationalen Forschenden – und zugleich Anfechter*innen des politischen Missbrauchs der Psychiatrie – betont. Siehe hierzu z. B. Klassiker aus der Zeit wie Bloch & Reddaway (1977) oder spätere Werke wie Reddaway (2020) und natürlich van Voren (2010).

4 Eine solche Liste, die insgesamt 103 Namen von Ärzt*innen, Pfleger*innen und Gesundheitsbeamt*innen umfasst, wurde etwa durch Alexander Podrabinek zusammengetragen und 1979 in New York als Teil seines Buchs *Карательная Медицина* (Strafmedizin) veröffentlicht. Eine noch detailliertere Liste über 288 Namen entstammt der kollektiven Autorschaft von Alexander Podrabinek, Felix Serebrov, Viacheslav Bakhmin und Leonard Ternovsky. Undatiert als Kopie im Archiv des Sacharow-Zentrums in Kaunas lagernd, muss dieses Dokument vom Anfang der 1980er Jahre stammen (Dokument der Arbeitskommission zur Untersuchung des Gebrauchs der Psychiatrie zu politischen Zwecken [Index der Ärztenamen] im Sacharow-Zentrum für demokratische Entwicklung, Vytautas-Magnus-Universität Kaunas).

5 Die Weltpsychiatrieassoziation wurde dabei zum Hauptschauplatz der Auseinandersetzung über den institutionalisierten Psychiatriemissbrauch in der Sowjetunion (eine sowjetische Stellungnahme dazu erschien dann in *Korsakov*, 1978, Bd. 78, Nr. 4: 607–625). Als die WPA 1977 eine Verurteilung sowjetischer Praktiken und die Declaration of Hawaii – einen Ethikcode fürs Fach – herausbrachte, wurde der politische Missbrauch der Psychiatrie auch beim Regionalsymposium der WHO thematisiert (siehe Twenty-Seventh Session of the Regional Committee for Europe. Report by the Regional Director. 10 November 1977. *World Health Organization*, EB61/11: 1). Ein inner- und außerhalb des Fachs breit zirkuliertes Programmstatement der WPA stammt aus dem Jahr 1982: *The Issue of Abuse* befasst sich explizit und detailliert mit dem Verlauf der schwierigen und im Endeffekt fruchtlosen Versuche dieser Organisation, eine offene Diskussion mit sowjetischer Seite zu führen (World Psychiatric Association, *The Issue of Abuse 1977–1983*, Bericht vom November 1982, zirkuliert an alle Mitgliedsgesellschaften, Executive Committee und Committee members, im Sacharow-Zentrum für demokratische Entwicklung, Vytautas-Magnus-Universität Kaunas).

6 Z. B. DOSMED, eine der IPSS folgende Studie zu Determinants of Outcome of Severe Mental Disorders (Wu 2021: 142).

7 Für die internationale Wissenszirkulation zwischen deutsch- und englischsprachigen psychiatrischen Communities der Zeit siehe Roelcke et al. (2010). Für die deutschsprachige Psychopolitik der Zeit siehe Freis (2019) bzw. Schmitt (2018: 61–71). Für die Einengung der internationalen Kooperation für sowjetische Psyche-bezogene Disziplinen (um die Zeit stark bei Physiologie und Genetik angesiedelt) siehe z. B. Krementsov (2005).

8 Siehe z. B. primär auf die britische Antipsychiatriebewegung fokussiert Wall (2018) bzw. Lienhard (2022). Zur Rezeption und Auseinandersetzung mit der Antipsychiatriebewegung in der BRD-Psychiatrie siehe u. a. Schmitt (2018: 333–401). Aus der Zeit selbst und die Bewegung mittragend siehe beispielsweise Foudraine (1971) und Szasz (1976).

9 Es ist beinahe unmöglich, eine allseits befriedigende und repräsentative Auswahl dieses Forschungsfeldes zu treffen. Exemplarisch seien daher erwähnt: Roer & Henkel (1996), Czech (2007), Yurdakul (2021).

10 Für den psychiatrischen Missbrauch zu politischen Zwecken in anderen staatssozialistischen Ländern siehe am Beispiel der DDR Sonja Süß (1998).

11 In dem Fall handelt es sich um eine Revision, die als vierte und letzte vor dem Zweiten Weltkrieg auf die Zeit vor der WHO-Gründung zurückgeht. Ab dem als Gründungsmoment angesehenen Jahr 1893 wurde die Klassifikation bis zum Ersten Weltkrieg durch das Internationale Statistische Institut, in der Zwischenkriegszeit durch eine Mischkommission aus diesem Institut und der Gesundheitsorganisation des Völkerbundes, nach dem Zweiten Weltkrieg durch die neu entstandene WHO kuratiert, immer wieder neu aufgelegt – und durch die Herausgeberautorität legitimiert.

12 Zitiert aus der 1948 gedruckten deutschsprachigen Fassung der 5. Revision von 1938 (Statistisches Zentralamt 1948: 15).

13 In Diem (1903) als *dementia simplex*, „die einfach demente Form der Dementia praecox“: 111.

14 Der primäre Unterschied bestand hauptsächlich in der außerpsychiatrischen Konstruktion der Mainstreamgesellschaft, von der die Erkrankten im geringeren und für Unbefugte kaum sichtbaren Ausmaß abwichen. Wo für Diem die Verflachung der Affekte sich in einem leichtem Sozialabstieg im bürgerlichen Rahmen äußerte – zu einfacheren Berufsposten etwa (Diem 1903: 111) –, trat diese für Snezhnevsky beispielsweise in einer unbegründbaren Begeisterung eines vermeintlich Erkrankten für Yoga bzw. im Wunsch, zu spirituellen Zwecken nach Indien zu reisen, zutage (Snezhnevsky und Yastrebov 2019: 33–34).

15 Im Fall Kerbikovs handelt es sich um eine kollaborative Klassifikation psychiatrischer Erkrankungen eines Forscher*innenteams, dem auch Andrey Snezhnevsky angehörte. Die eigene auf Schizophrenie spezifizierte Klassifikation wurde von Snezhnevsky samt dem theoretischen Rahmen zu Ätiologie und Verlauf in denselben Jahren erarbeitet.

16 Die ursprünglich im Jahr 1957 erschienenen *Skizzen zur Geschichte heimischer Psychiatrie* Dmitry Fedotovs beharren mehrfach darauf, dass die „russische“ (sic!) Psychiatrie und Medizin immer humaner und origineller waren als „ausländische“ Ansätze und dass die russische Psychiatrie zwar von europäischen Schulen lernte, im Grunde aber immer etwas Eigenständiges und Unabhängiges war. Diese Idee ist belegbar falsch und schon allein wegen ihres isolationistischen Ansatzes und unselektiven Gebrauchs der Pars-pro-toto-Begriffe wie „russisch“ für vermeintlich „sowjetisch“ kritisch zu hinterfragen. Snezhnevsky zeigte sich etwa ein Jahrzehnt später viel moderater und bezüglich der internationalen Einordnung der sowjetischen Psychiatrie einsichtiger. Seine Argumentation suchte gerade umgekehrt das eigens erschaffene Schizophreniemodell durch universal europäische Wurzeln zusätzlich zu stärken.

17 Unter diesem Sentiment verstehe ich hier weniger die sowjetmarxistische Epistemologie, sondern eher das fortgeführte Narrativ, das sich sowohl in Budilova als auch (besonders stark) in Fedotov wiederfindet. Die eigentlich wissenschafts- bzw. medizinhistorische Erzählung wird in einen Diskurs eingeflochten, in dem die russischsprachige Medizin nicht als Bestandteil größerer europäischer Episteme, sondern als ein überragendes Unikum betrachtet wird. Es ändert sich dabei lediglich die Schattierung der Darstellweise: Während Fedotovs Buch, erstveröffentlicht im Jahr 1957, das Autochtone gegen das „Fremde“ in der Psychiatrie nahezu feindselig herausstreicht, lamentiert Morozov (2016) nur en passant über mangelnde Kenntnisse im Ausland russischsprachiger Psychiater*innen aus dem 19. Jahrhundert.

18 Im Übrigen wird Kronfeld in seinen letzten in der UdSSR verbrachten Lebensjahren immer wieder als Mentor Snezhnevskys angeführt (Shifrin 2017).

19 Nicht einmal Anatoly Koriagin, selbst ein Psychiater, der sich dem Usus politischer Fehldiagnosen widersetzte, mit der Dissident*innenbewegung aktiv kooperierte und dafür zu über sieben Jahren Lager und Verbannung verurteilt wurde, legte Snezhnevsky solch bösartige Absicht zur Last (siehe Koriagin in Posev 1987, 12: 46).

20 Obwohl die DDR-Psychiatrie ihre eigene und wahrscheinlich die weltweit ausdifferenzierteste Klassifikation der Schizophrenie – nach Leonhard – hatte und auch in Einzelfällen des politischen Missbrauchs des Fachs beschuldigt wurde (für die DDR ist dieses Thema am besten durch Sonja Süß 1998 ausgearbeitet), wurden weder Karl Leonhard noch seine Klassifikation jemals in Zusammenhang mit der Unterdrückung der Menschenrechtsbewegung gebracht, wie dies für Snezhnevsky und sein Modell der Fall ist.

21 WHO, Report of the international conference for the ninth revision of the International Classification of Diseases, Dokument A29/32, 22.04.1976.

22 Es sei an dieser Stelle angemerkt, dass es nur drei WHO-eingeleitete Adaptionen der ICD‑9 gab: je eine für Onkologie, Augen- und Zahnheilkunde. Der Begriff „Adaptation“ ist also in Bezug auf die sowjetische Ausgabe des Kapitels V problematisch, war sie doch nicht von der WHO sanktioniert und leicht an landesübliche Benennungen angepasst, sondern als stark revidierte neue Adaptation von und für die Sowjetunion gemacht.

23 WHO, Manual of the International Statistical Classification of Diseases, Injuries, and Causes of Death (russische Fassung bei selbem Dokumenttitel, aus dem Russischen übersetzt): Vorwort ohne Seitennummerierung.

24 Für die 1970er Jahre weisen die mir vorliegenden deutschsprachigen Ausgaben des original russischen Psychologiehandbuchs von Arthur Petrowski (Auflage 1986; nach 1974 erfolgten Übersetzung vom Originaltext aus dem Jahr 1970) und die dritte Auflage des US-amerikanischen Klassikers von Zimbardo & Ruch (1978) allein anhand ihrer Inhaltsverzeichnisse eine durchaus große Themenkonvergenz auf, die bei allen Verschiedenheiten der Zugänge dennoch die Einschätzung zulässt, dass die Psychologie in der Sowjetunion halbwegs dieselben disziplinären Grenzen und Forschungsgegenstände hatte wie anderswo auf der Welt. Abgesehen davon ist die zeitgenössische – etwa klinische – Psychologie durchaus mit Diagnostikaufgaben betraut, bei denen das Instrumentarium der ICD zur Anwendung kommt.

25 ICD‑9, englischsprachige Version von 1977: 218, russischsprachige WHO-Fassung: 179, sowjetische Ausgabe von 1983: 30, bzw. deutschsprachige Fassung der DDR 1983: 267.

26 Snezhnevsky selbst führt in den einleitenden Worten zu seinem Handbuch *Schizophrenie. Multidisziplinäre Studie* einige uns bereits bekannte Formen an, die er in der argumentativen Logik vollkommen synonym verwendet. Wo er von einem wegen Stressfaktoren im familiären Umfeld Schizophrenie-vulnerablen „Persönlichkeitstyp“ spricht, finden wir in seinem Text folgende Passage: „In der Psychiatrie existiert ein mehr oder weniger glücklicher Begriff für verschiedene Varianten vom beschriebenen Persönlichkeitstyp und der Bezeichnung, die von einigen als ‚tiefe Schizoidie‘, von anderen als ‚latente Schizophrenie‘ oder ‚residuale Schizophrenie‘ bezeichnet wird“ (1972: 6).

27 Im Original: „латентная шизофрения (вялотекущая, малопрогредиентная)“.

28 Sowjetische Fassung der 9. Revision der ICD (aus dem Russischen übersetzt), 1983: 28.

29 Brief von Alexander Voloshanovich an Peter Sainsbury bzgl. des Falls Klebanov von 01.01.1982, im Sakharov Center for Democratic Development, Kaunas.

30 Sowjetische Fassung der 9. Revision der ICD, 1983: 29.

31 Sowjetische Fassung der 9. Revision der ICD, 1983: 6.

32 Report of the International Pilot Study of Schizophrenia. Bd. 1, WHO (1973: 168).

33 Dies ist eine Schlussfolgerung, die an keiner Textstelle im Bericht erläutert wird. Zu diesem Schluss komme ich durch die idente Zahlenangabe der Patient*innen mit dieser Subform – einerseits an der Stelle, wo das IPSS-Leitungsteam die begriffliche Divergenz anspricht (168) und andererseits an einer weiteren Stelle (Table 10.2. auf S. 163), wo diese kommentarlos als „latent“ bezeichnet wird.

34 Sowjetische Fassung der 9. Revision der ICD, 1983: 5.

35 WHO, 29. Session der Generalversammlung der Weltgesundheitsorganisation, Dokument A29/32 vom 22.03.1976.

36 Ebd: 40. Für das regionale Zentrum siehe auch die russischsprachige ICD‑9 Ausgabe der WHO, 1977: XXXVI.

37 Report on Appointment to Expert Advisory Panels and Committees by the WHO’s Executive Board of 20.12.1961 (EB29/32).

38 Report on Appointment to Expert Advisory Panels and Committees by the WHO’s Executive Board of 03.01.1972 (EB49/31).

39 *Reference Aid. Directory of Soviet Officials* (Bd. 1: *National Organizations*) der CIA von Dezember 1975: 142.

40 Twenty-Seventh Session of the Regional Committee for Europe. Report by the Regional Director. 10 November 1977. World Health Organization, EB61/11: 1.

41 Dieser Bericht ist als Anhang (No3043s) zu Dokument No3042s verfügbar; 21.11.1977 vom Gesundheitsminister Petrovsky ans Zentralkomitee. Bukovsky-Archiv online: https://bukovskyoldarchive.files.wordpress.com/2015/07/21-nov-77-9-pp-no-3042.pdf (letzter Zugriff 17.01.2023): 1–2.

42 Berichte zu dieser „Studienfahrt“ erschienen in einer zehnteiligen Reihe in der *Volksstimme*, der Zeitung der österreichischen kommunistischen Partei. Die Serie unter Autorschaft von Hans Wolker wurde für die Zwecke dieses Artikels im Sakharov Center for Democratic Development an der Vytautas-Magnus-Universität Kaunas konsultiert.

43 Neben den bereits erwähnten Personen Morozov, Babaian und Snezhnevsky waren auch Zharikov und Nadzharov in den Dissident*innenlisten der mit Missbrauch angelasteten, namentlich bekannten Ärzt*innen vertreten.

44 Mark Hurst verfasste eine erwähnenswerte rezente Veröffentlichung, die auf die Gründe dieser ersten und eigentlich auch recht zögerlichen internationalen Rezeption der bekanntwerdenden Fälle politisch motivierter Fehldiagnosen, -einweisungen und -behandlungen eingeht.

45 Wie z. B. bei WHO (1972: Titelblatt).
